# Evaluation of three biometric devices: ocular parameters and calculated intraocular lens power

**DOI:** 10.1038/s41598-022-24017-8

**Published:** 2022-11-14

**Authors:** Rivkah Lender, Devora Mirsky, Riki Greenberger, Zipora Boim, Lee Ben-Yaakov, Chaya Kashtan, Ibrahim Naffar, Shira Shine, Itay Chowers, Hadas Ben-Eli

**Affiliations:** 1 Department of Ophthalmology, Hadassah Hebrew University Medical Center, Jerusalem, Israel; 2 Department of Optometry and Vision Science, Hadassah Academic College, Jerusalem, Israel

**Keywords:** Diseases, Health care

## Abstract

Cataract surgery is among the most common medical procedures, and accurate ocular biometry measurements are key for successful visual outcome. The current study evaluated data obtained by the Eyestar 900, Anterion, IOLMaster700 biometers and the Pentacam corneal topographer. Compared values were axial length (AL), anterior chamber depth (ACD), steep- and flat-K, cylinder and axis. Clinical impact was assessed by calculating intraocular lens (IOL) power using the mean values of every parameter and the Barrett and Kane formulas, stratified by device and amount of cylinder. IOL was re-calculated for each device substituting Pentacam K-values. This study included 196 eyes (98 participants) of cataract surgery candidates. When comparing the IOLMaster to the Eyestar (157 eyes), no difference was found in mean AL or ACD measurements (P > 0.05). Steep-K measurements differed between these devices and the Pentacam (P = 0.01). AL and ACD measurements differed between the IOLMaster and Anterion (38 eyes; P < 0.05). Strong correlations (range 0.72–0.99) were found between all four devices. Bland–Altman analysis demonstrated excellent agreement between biometry devices other than ACD between the IOLMaster and Eyestar. Calculated IOL power was 0.50–1.00 diopter (D) lower with the IOLMaster. Cylinder power was 0.75D higher in all biometers when Pentacam K-values were substituted.

## Introduction

Cataract surgery, in which the opaque crystalline lens is replaced with an artificial intraocular lens (IOL), is one of the most common and successful medical procedures performed worldwide^1,2^. Ocular biometric parameters are measured before surgery in order to calculate the optimal IOL for the individual patient. The axial length (AL) of the eye, anterior chamber depth (ACD) and corneal curvature (K) measurements strongly impact the calculated IOL^3^. Accurate pre-operative biometric measurements are considered one of the most important factors for successful surgical result^4^, especially with the evolution of toric and multifocal IOLs which require the utmost surgical precision. As new or updated biometric devices are brought into clinical practice, it is important to thoroughly understand their capabilities, agreement with existing devices, and impact on IOL selection.

Swept-source optical coherence tomography (SS-OCT) is a non-invasive imaging modality that scans biological structures at high resolution, speed and sensitivity. The output volume of scans enables three-dimensional analysis of the given structure^5^. Anterior segment OCT (ASOCT) scans the cornea, sclera and lens, thereby providing vital information for IOL selection in cataract surgeries. The Eyestar 900 (Haag Streit Diagnostics, Koeniz, Switzerland) and Anterion (Heidelberg Engineering, Heidelberg, Germany) are two recently developed biometers based on the SS-ASOCT technology. In contrast, the Pentacam corneal tomographer (Oculus Inc., Wetzlar, Germany) measures the K values using a rotating Sheimpflug camera that acquires cross-sectional scans of the cornea^6^. In the clinic, when biometric devices fail to produce K-readings due to highly irregular corneas, corneal topography values are obtained and used to calculate the suggested IOL. While several studies evaluating the ocular measurements have already shown good repeatability and agreement between established biometers^7,8^, differences in specific parameters such as anterior chamber depth (ACD) and corneal curvatures (K) have been reported relative to SS-ASOCT devices such as the IOLMaster 700 and corneal tomographers such as the Pentacam^9–11^. Additionally, there is limited data in the literature as to whether these differences have any clinical impact, i.e., whether they affect the final selection of IOL^12,13^. The purpose of this study was therefore to compare the ocular parameters measured by these two recent biometers to those measured by the IOLMaster 700 (Carl Zeiss Meditec, Jena, Germany), the first SS-OCT-based biometric device and one of the most commonly used devices for pre-cataract surgery measurements^14^. To further investigate the accuracy of SS-OCT corneal measurements, these values were also compared to those obtained by the Pentacam (Oculus, Wetzlar, Germany) topographer. The final lens power calculations based on the measurements of each biometric device were compared to examine the clinical impact.

## Methods

This retrospective study followed the tenants of the Helsinki declaration and was approved by the institutional Helsinki Committee of Hadassah Medical Center (study number HMO-0459-18). The committee exempts retrospective research from informed consent by the participants. Data was collected from the Ophthalmology Department database and anonymized before analysis. Before biometric measurements, each participant underwent ophthalmic examination including best-corrected visual acuity (BCVA) with and without pinhole and full slit-lamp evaluation.

### Ocular measurements

Identical ocular parameters were measured using three different ocular biometry devices: the The Eyestar 900 (Haag Streit Diagnostics, Koeniz, Switzerland), the Anterion (Heidelberg Engineering, Heidelberg, Germany) and the IOLMaster 700 (Carl Zeiss Meditec, Jena, Germany). The parameters compared were axial length (AL), anterior chamber depth (ACD), anterior corneal values (steep- and flat-K), cylinder (cyl) and axis. Additionally, K values were compared to those obtained by the Pentacam corneal tomographer (Oculus Inc., Wetzlar, Germany). Measurements in all four devices were performed by optometrists with both the participants’ eyes open during each measurement. In cases where more than one measurement was performed for a single participant using the same instrument, only the most recent complete measurement was included in the final analysis.

The parameters included in this study have the greatest impact on IOL calculation. Therefore, in order to assess whether any discrepancies between the different devices would result in differences in the recommended IOL, the mean values of every parameter for each biometry device were entered in the Barrett Universal II Formula^15^, Barrett Toric Calculator^16^ and Kane Formula ^17^ and the final IOLs were compared. Monofocal IOL formulas were used for eyes with less than 1.00 diopter (D) cylinder, and toric IOL formulas were used for eyes with ≥ 1.00D cylinder. Since corneal topographers are commonly used in cases where biometers fail to provide the K-values, such as very steep or distorted corneas, IOL calculations were repeated with the Pentacam K-values substituting the different biometers’ K-values in order to assess the impact on the final power of the suggested IOL.

### Participants

All participants included in this study were cataract surgery candidates who presented at the clinic for standard biometric measurements before surgery. Excluded were participants under age 40, with missing study parameters, and/or whose ocular measurements had a standard deviation greater than 0.05 (mm or D). Participants were measured using the Eyestar 900 and IOLMaster 700 (Group A) or the Anterion and IOLMaster 700 (Group B). A subset of each group had corneal values measured also with the Pentacam. Participants were also stratified by amount of cylinder, with a cut-off level of 1.00D.

### Sample size

Assuming average absolute difference between devices of 0.024 mm for AL with SD of 0.05 mm to the greatest difference in mean measurements^18^, the minimal paired sample size that is required is 73 eyes for each group to achieve a power of 80% and a level of significance of 5%, two sided, using the *statulator* sample size calculator.

### Statistical analysis

The normality of the data was confirmed using the Shapiro–Wilk test. AL, ACD, flat- and steep-K, cylinder and axis were compared using paired sample t-test or Friedman test, according to the sample size. Cohen's *d* was performed on values that were significantly different in pairwise analysis in order to examine effect size and clinical significance. Correlation between parameters measured by different devices was evaluated with Pearson's coefficient. Mean values of AL, ACD and K’s of each device were used to calculate the recommended IOL by the Barrett Universal II Formula, Barrett Toric and the Kane calculators. IOL power was calculated using the mean parameters of each biomter and stratified by device and amount of cylinder. Bland and Altman analysis was used to identify systematic agreement between the measurements by the different devices and to identify outliers. Linear regression was used to assess the significance of agreement level between biometry devices. The analysis was performed using SPSS software (IBM SPSS Statistics, Version 27.0, Chicago. Armonk, NY: IBM Corp).

### Ethical considerations

The institutional Helsinki committee exempts retrospective research from informed consent by the participants.

## Results

### Patient characteristics

Records of 238 eyes (119 participants) were extracted from the Eyestar 900 and Anterion devices. This data was filtered for the current study based on age and data completeness, resulting in the exclusion of 42 eyes (17.6%). Ultimately, a total of 196 eyes (98 participants, 47.9% female, mean age 68.8 ± 9.7 years, range 42–88 years) were included in this study. Participants were divided to two groups: Group A consisted of 157 eyes (79 participants, 80.6%) measured with the Eyestar and IOLMaster, with 48 eyes (24.4%) also measured by Pentacam. Group B consisted of 38 eyes (19 participants, 19.3%) measured with the Anterion and IOLMaster, with 22 eyes (11.2%) also measured by Pentacam. Table 1 describes the demographic characteristics of the study population.

**Table 1:** Basic characteristics of study population.

	Group A	Group B	P
N eyes (participants)	157 (79)	38 (19)	NA
Female N (%)	78 (49.0)	16 (42.1)	0.42^a^
Mean age ± SD (years)	68.7 ± 10.0	69.1 ± 8.7	0.80^b^
Age range (years)	42.0–88.0	48.0–82.0	NA
Eyestar 900	157 eyes	NA	NA
IOLmaster 700	157 eyes	38 eyes	NA
Anterion	NA	38 eyes	NA
Pentacam	48 eyes	22 eyes	NA

^a^Chi-Square.

^b^Independent sample t-test.

### Biometry results

In Group A there was no difference in mean AL values when comparing the IOLMaster (24.22 ± 2.0 mm) to the Eyestar 900 (24.2 ± 2.04 mm; P = 0.79; Cohen’s d 0.009). Mean ACD was also similar (3.34 ± 0.56 mm vs. 3.36 ± 0.56 mm, respectively; P = 0.09; Cohen’s d 0.03). No differences were found in mean flat and steep K values or in cylinder axis (Table 2). A sub-group of 48 eyes in Group A were also measured using the Pentacam. In this comparison, a difference was found in mean steep K measurements between the IOLMaster (45.02 ± 1.84D), the Eyestar (44.99 ± 1.85D) and the Pentacam (44.88 ± 1.76D) (P = 0.01). No differences were found in mean flat K values or in cylinder axis (Table 2).

**Table 2:** Biometric parameters—mean differences (Group A).

	IOLmaster700 Mean ± SD	Eyestar900 Mean ± SD	Pentacam Mean ± SD	P^a^	Cohen's *d*
N	**157**	**157**	***N/A***		
AL (mm)	24.22 ± 2.00	24.20 ± 2.04	*N/A*	0.79	0.009
ACD (mm)	3.34 ± 0.56	3.36 ± 0.56	*N/A*	0.09	0.03
Flat K (D)	43.23 ± 2.07	43.22 ± 2.09	*N/A*	0.64	0.004
Steep K (D)	44.52 ± 1.92	44.51 ± 1.95	*N/A*	0.65	0.005
Cyl (D)	1.31 ± 1.34	1.32 ± 1.33	*N/A*	0.61	0.007

				P^b^	
N	**48**	**48**	**48**		
Flat K (D)	43.12 ± 1.79	43.14 ± 188	43.17 ± 1.71	0.67	*N/A*
Steep K (D)	45.02 ± 1.84	44.99 ± 1.85	44.88 ± 1.76	**0.01**	*N/A*
Cyl (D)	1.93 ± 1.66	1.92 ± 1.68	1.72 ± 1.50	0.16	*N/A*
Flat K axis (°)	83.17 ± 59.92	79.91 ± 57.74	89.59 ± 55.80	0.67	*N/A*
Steep K axis (°)	76.12 ± 54.12	80.47 ± 53.56	89.91 ± 58.51	0.25	*N/A*

*ACD* anterior chamber depth, *AL* Axial length, *Cyl*: cylinder.

^a^Paired sample t-test.

^b^Friedman test.

Significant values are in [bold].

In Group B, mean AL values were different when comparing the IOLMaster to the Anterion (23.68 ± 1.27 mm vs. 23.57 ± 1.27 mm, respectively; P = 0.006; Cohen’s d 0.08). Mean ACD was also different (3.27 ± 0.73 mm vs. 3.38 ± 0.78 mm; P = 0.04; Cohen’s d 0.14). No differences were found in mean flat and steep K values or in cylinder axis (Table 3). A sub-group of 22 eyes in Group B were also measured using the Pentacam. No significant differences were found in any corneal parameter between the IOMaster, Anterion and Pentacam (Table 3).

**Table 3:** Biometric parameters—mean differences (Group B).

	IOLmaster700 Mean ± SD	Anterion Mean ± SD	Pentacam Mean ± SD	P^a^	Cohen's *d*
N	**38**	**38**	***N/A***		
AL (mm)	23.68 ± 1.27	23.57 ± 1.27	*N/A*	**0.006**	0.08
ACD (mm)	3.27 ± 0.73	3.38 ± 0.78	*N/A*	**0.04**	0.14
Flat K (D)	43.73 ± 1.94	43.61 ± 1.83	*N/A*	0.19	0.06
Steep K (D)	45.51 ± 1.75	45.40 ± 1.97	*N/A*	0.51	0.05
Cyl (D)	1.77 ± 1.64	1.78 ± 1.79	*N/A*	0.94	0.005

				P^b^	
N	**22**	**22**	**22**		
Flat K (D)	43.33 ± 2.02	43.30 ± 1.96	43.45 ± 2.10	0.46	*N/A*
Steep K (D)	45.30 ± 2.11	45.33 ± 2.38	45.33 ± 2.38	0.23	*N/A*
Cyl (D)	1.96 ± 1.64	2.08 ± 1.88	1.82 ± 1.77	0.59	*N/A*
Flat K axis (°)	104.13 ± 61.34	90.81 ± 62.48	108.22 ± 59.62	0.31	*N/A*
Steep K axis (°)	79.59 ± 45.66	74.45 ± 45.17	73.63 ± 44.44	0.09	*N/A*

*ACD* anterior chamber depth, *AL* Axial length, *Cyl* cylinder.

^a^Paired sample t-test.

^b^Friedman test.

Significant values are in [bold].

### Analysis of correlations and agreements across devices

When testing the correlations between the different devices, strong correlations were found between all four devices in AL, ACD, flat and steep K values and cylinder, ranging from R = 0.72 to 0.99. (Table 4) Comparison of cylinder axes between the different devices yielded weaker correlations, ranging from R = 0.32 to 0.95 (Table 4).

**Table 4:** Matrix of correlations.

Parameter	Devices	r	P
AL	IOLMaster700-Anterion	0.99	< 0.001
	IOLMaster 700-Eyestar900	0.88	< 0.001
ACD	IOLMaster700-Anterion	0.93	< 0.001
	IOLMaster 700-Eyestar900	0.90	< 0.001
Flat K	IOLMaster700-Eyestar	0.97	< 0.001
	IOLMaster700-Anterion	0.95	< 0.001
	IOLMaster700-Pentacam	0.95	< 0.001
	Eyestar-Pentacam	0.91	< 0.001
	Anterion-Pentacam	0.89	< 0.001
Flat K axis	IOLMaster700-Eyestar	0.67	< 0.001
	IOLMaster700-Anterion	0.39	0.01
	IOLMaster700-Pentacam	0.65	< 0.001
	Eyestar-Pentacam	0.51	< 0.001
	Anterion-Pentacam	0.32	0.13
Steep K	IOLMaster700-Eyestar	0.99	< 0.001
	IOLMaster700-Anterion	0.86	< 0.001
	IOLMaster700-Pentacam	0.97	< 0.001
	Eyestar-Pentacam	0.98	< 0.001
	Anterion-Pentacam	0.85	< 0.001
Steep K axis	IOLMaster700-Eyestar	0.64	< 0.001
	IOLMaster700-Anterion	0.88	< 0.001
	IOLMaster700-Pentacam	0.39	0.001
	Eyestar-Pentacam	0.36	0.01
	Anterion-Pentacam	0.95	< 0.001
Cyl	IOLMaster700-Eyestar	0.96	< 0.001
	IOLMaster700-Anterion	0.82	< 0.001
	IOLMaster700-Pentacam	0.95	< 0.001
	Eyestar-Pentacam	0.97	< 0.001
	Anterion-Pentacam	0.72	< 0.001

*ACD* anterior chamber depth, *AL* Axial length, *Cyl* cylinder.

Bland and Altman analysis was performed in order to assess the agreement between the biometry devices. A preliminary analysis demonstrated no difference between the IOLMaster and Eyestar and therefore AL, ACD, and flat and steep K values were all included in the agreement analysis. However, a difference between the IOLMaster and Anterion in mean AL and ACD values (P = 0.006, P = 0.04; respectively) was observed. Because these parameters did not show a useful level of agreement, there were not included in the Bland and Altman analysis.

**Figure 1:**
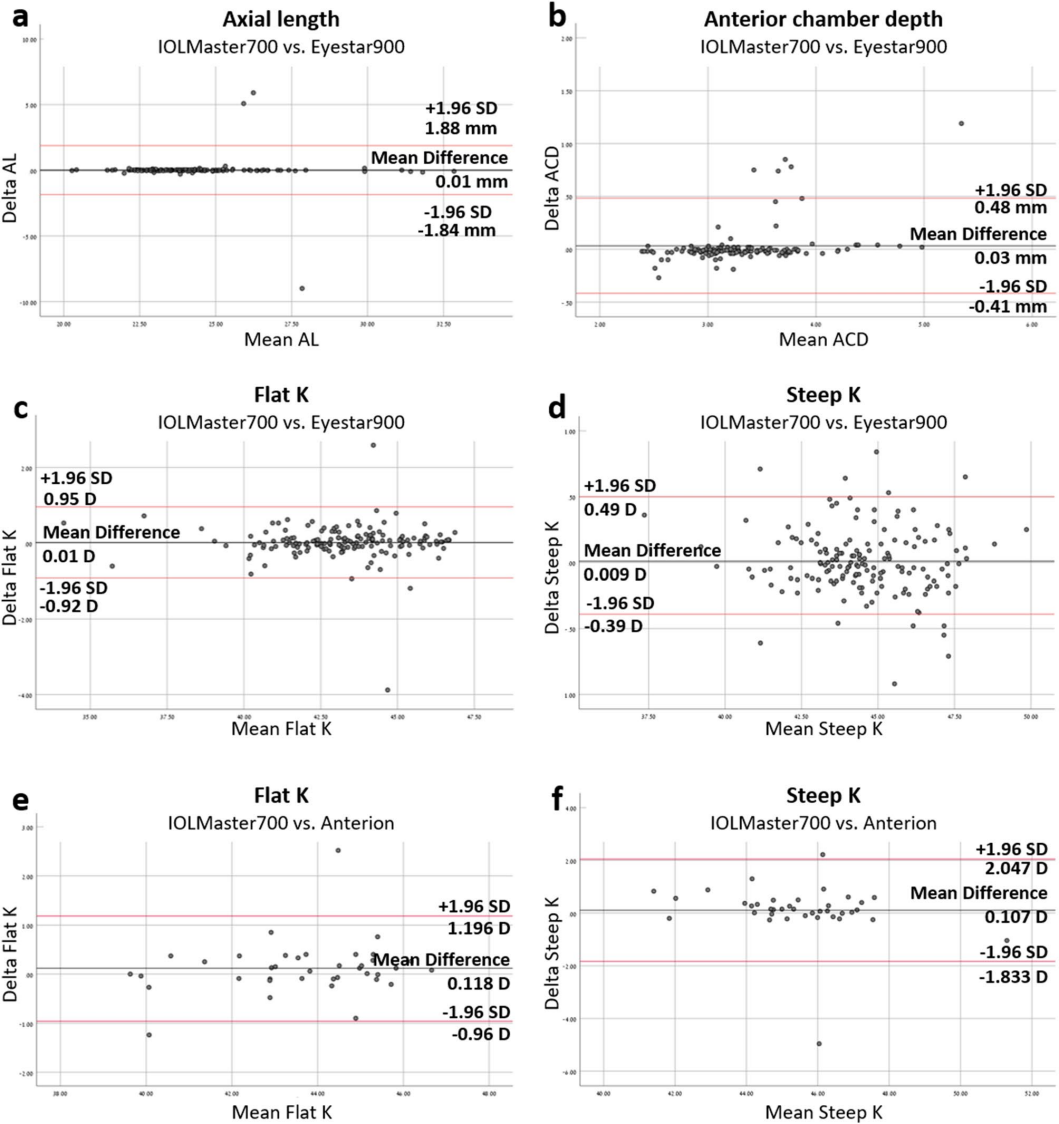
Bland and Altman agreement analysis of IOLMaster700, Eyestar900 and Anterion. Bland and Altman analysis of axial length, anterior chamber depth and flat and steep K in the IOLMaster vs. the Eyestar (**a**–**d**), and flat and steep K in the IOLMaster vs. the Anterion (**e**,**f**). Axial length and anterior chamber depth are measured in millimeters, and flat and steep K in diopters. (**a **) No significant difference between IOLMaster and Eyestar scatter dots in axial length measurements, P = 0.54. (**b**) Significant difference between IOLMaster and Eyestar scatter dots in anterior chamber depth measurements, P < 0.001. (**c**) No significant difference between IOLMaster and Eyestar scatter dots in flat K measurements, P = 0.69. (**d**) No significant difference between IOLMaster and Eyestar scatter dots in steep K measurements, P = 0.21. (**e**) No significant difference between IOLMaster and Anterion scatter dots in flat K measurements, P = 0.23. (**f**) No significant difference between IOLMaster and Eyestar scatter dots in steep K measurements, P = 0.18. *D* Diopters, *ACD* anterior chamber depth, *AL* Axial length.

When analyzing the linear regression line of the Bland and Altman scatter plot, IOLMaster and Eyestar values demonstrated no difference of proportional bias of scatter dots above and below the mean difference line for AL and flat and steep K values (P = 0.54, P = 0.69, P = 0.21, respectively). These devices differed in ACD values (P < 0.001), with a higher amount of ACD observations above the mean difference line. IOLmaster and Anterion flat and steep K plots were equally distributed above and below the mean difference line (P = 0.23, P = 0.18, respectively) (Fig. 1).

### Effect of biometry measurements on IOL power calculation

In order to investigate whether the minor differences observed between the devices impacted the suggested IOL power, the mean output values of each device (Tables 2 and 3) were inserted to the Barrett Universal II Formula, Barrett and Kane toric online calculators. Parameters were consistent between calculators: Bausch & Lomb MX60 and MX60T lenses, A-Constant 119.15, surgical induced astigmatism (SIA) 0, target refraction plano, and incision location at 100° for right eye of each IOL calculation.

When comparing the IOLMaster and Eyestar (Group A) using the Barrett toric calculator, the calculated IOLs were 18.50/2.00D and 19.00/2.00D, respectively. Using the Kane toric calculator, both IOLs were 19.00/2.25D. In Group B, comparison of the IOLMaster and Anterion with the Barrett toric calculator yielded calculated IOLs of 19.50/2.75D and 20.00/2.75D, respectively, and with the Kane toric calculator 20.00/2.25D and 20.50/2.25D, respectively. It can be seen that the power of the suggested IOL based on the Barrett toric calculator was 0.50D lower in the IOLMaster relative to the Eyestar, but no difference was found using the Kane toric calculator. This same 0.50D difference was again found with both calculators when comparing the IOLMaster and Anterion. (Table 5) IOL calculation was then repeated with Pentacam K-values inserted in place of the K-values of each device. This resulted in a consistent increase of 0.75D in the cylinder power in all biometers with both formulas (Table 5).

**Table 5:** Suggested IOL power (diopters) stratified by device.

	Formula	Group A N = 157		Group B N = 38	
		IOLmaster700	Eyestar	IOLMaster700	Anterion
Biometer values	Barrett Toric	18.50/2.00	19.00/2.00	19.50/2.75	20.00/2.25
	Kane Toric	19.00/2.25	19.00/2.25	20.00/2.25	20.50/2.25
Pentacam K-values	Barrett Toric	18.50/2.75	18.50/2.75	20.00/3.50	20.00/3.50
	Kane Toric	19.00/3.00	19.00/3.00	19.00/3.00	20.50/3.00

Biometer values: IOL calculations were performed using the Barrett and Kane toric calculators with values obtained by the different biometry devices.

Pentacam K-values: The calculations were repeated with the biometers’ K values replaced by the Pentacam K values.

Table 6 describes the calculation of suggested IOL power stratified by amount of cylinder. The cut-off was 1.00D cylinder; eyes with < 1.00D were classified as ‘low’ cylinder and monofocal IOL formulas were used to calculate the suggested IOL. Eyes with ≥ 1.00D cylinder were classified as ‘moderate-high’ cylinder and toric IOL formulas were used. In the Group A low cylinder eyes, it was found that all suggested monofocal IOLs were identical across both biometers and formulas. However, in the moderate-high cylinder eyes, the same trend observed in the non-stratified calculation was again apparent, with the IOLMaster yielding a 0.50D lower power in the suggested IOL. In the Group B low cylinder eyes, the suggested power was 0.50–1.00D lower with the IOLMaster than the Anterion. In the moderate-high cylinder eyes, the suggested IOL power was 0.50D lower with the IOLMaster than the Anterion when applying the Barrett toric calculator.

**Table 6:** Suggested IOL power (diopters) stratified by cylinder amount.

	Formula	Group A		Group B	
		IOLmaster700	Eyestar	IOLMaster700	Anterion
Low cylinder (< 1.00D)		N = 88		N = 14	
	Barrett Universal II	19.50	19.50	20.00	20.50
	Kane	19.50	19.50	20.00	21.00
Moderate—high cylinder (≥ 1.00D)		N = 69		N = 24	
	Barrett Toric	18.00/3.50	18.50/3.50	19.50/4.25	20.00/4.25
	Kane Toric	18.00/3.00	18.50/3.00	20.00/3.75	20.00/3.75

## Discussion

The primary aim of this study was to compare ocular parameters measured by three recent biometers and assess the effects of any difference on the calculated IOL power for implantation in cataract surgery. The biometers’ K-values were also compared to K-values measured by a Scheimpflug-based corneal topographer.

When comparing AL and ACD measurements, a statistically significant difference was found between IOLMaster and Anterion but not between the IOLMaster and Eyestar. However, Cohen’s *d* analysis demonstrated that these differences were not clinically significant, and all values were highly correlated. These results are in line with recent findings by Fişuş and collaborators, which revealed discrepancies between measurements of the Anterion and IOLMaster^9^. Similarly, Tañá-Rivero et et al. also reported shorter ACD measurements with the IOLMaster relative to the Anterion^10^. Like in the current study, the differences in measurements were found to be statistically significant but were minor enough to most likely be clinically insignificant.

In addition to comparing between the different biometry devices, this study also compared K-readings of the three biometers to those of the Pentacam corneal topographer. There is clinical importance in understanding the correlation between the two modalities, because biometers can be limited in their ability to produce K-readings in cases of highly irregular corneas. In such cases, these values are often extracted from a corneal topographer or keratometer. No differences were found in the current study between any of the biometers in flat and steep K values or axes, yet when comparing these values to the Pentacam measurements, a statistically significant difference was observed between the IOLMaster, Eyestar and Pentacam in steep K values. Interestingly, the flat and steep K values were strongly correlated between all devices while axis values demonstrated low to medium correlations. Fişuş et al. did report significant differences in flat and steep K values^9^, but this may be due to differences in sample size. This is supported by the results presented by Tañá-Rivero et al., which did not find significant differences in K-values and were based on a sample size similar to that of the current study^10^. The matter of K axes has not yet been sufficiently studied and must be further investigated, as accurate axis measurements are vital for successful toric lens implantation.

In order to establish interchangeability of the devices, Bland and Altman analysis was performed. Our results indicated good agreement between all three biometers on most parameters, with a minor offset in ACD measurements between the IOLMaster and the Eyestar. Although Fişuş et al. advised against using the Anterion and IOLMaster interchangeably^9^, the lack of clinically significant differences between the devices leads us to suggest that these two devices can be used interchangeably, between themselves as well as with the Eyestar.

An additional aspect of the primary aim was to evaluate the clinical impact of the differences between the devices. This was achieved by calculation of the suggested IOL power based on the mean values of each device, and re-calculating with the substitution of Pentacam K-readings. Applying two commonly used IOL calculators to the entire study group, i.e., with no stratification by cylinder amount, the IOLMaster tended to yield a suggested IOL with 0.50D lower power relative to the Eyestar and Anterion. Tañá-Sanz et al. and Shetty et al. reported differences in the calculated IOL between the IOLMaster and Anterion which were statistically significant but clinically insignificant^12,13^. As the post-cataract surgery refractive targets suggested by the Royal College of Ophthalmology are within ± 1.00D for 85% of patients and within ± 0.50D for 55% of patients^19^, a difference of 0.50D can be viewed as clinically insignificant. When Pentacam K-values were inserted into the IOL calculation, the cylinder power was consistently 0.75D higher across all biometric devices. As mentioned previously, in a similar comparison Tañá-Rivero et et al. did not find significant differences in the K-values, however this study did not assess the effect on the selected IOL^10^. In a systematic review, Kane and Chang^18^ also found minor differences in measured values and calculated IOL. However, they emphasize that while devices may not differ statistically in the overall mean, they cannot be considered interchangeable due to the differences that can occur in the same eye between devices. Additionally, while this review concludes that differences between devices rarely affect the suggested IOL, there is almost no data on the Eyestar and Anterion devices that were assessed in the current study.

Further calculations were performed on sub-groups stratified by amount of cylinder. These analyses revealed differences between devices of up to 1.00D in the suggested IOL among the low cylinder eyes. While still technically acceptable according to the Royal College of Ophthalmology guidelines^19^, in an era where cataract surgeries can be considered refractive surgeries rather than just rehabilitative procedures, it is vital to take note of these differences and perform further studies to validate this preliminary data.

This study had several limitations, primarily due to the retrospective study design which strongly influenced the data available for analysis and caused limited and unequal sample sizes between groups. Additionally, because the study was not built prospectively, many viable measurements could not be included in the study since the patients had only undergone measurements by one biometric device. The Pentacam sub-groups and stratification by cylinder amount further limited the sample size, and therefore this data should be viewed as a preliminary basis for further research. It is worth noting that the IOLMaster and Pentacam were consistent in their biases relative to other devices in the suggested IOL power, despite the limited sample group. The main strength of this study, however, is the comparison of three biometry devices, two of which are quite recent with limited data in the literature. Moreover, the comparison of corneal measurements between SS-OCT devices and a Scheimpflug-based device further validates both technologies and also provides a quality assessment of these devices as well as clinical impact.

In conclusion, all three biometers compared in this study demonstrated strong agreement in their individual parameters, with corneal measurements correlating well to the Pentacam. However, it is important to note that the Eyestar was more similar to the IOLMaster in both AL and ACD, which are critical features in IOL calculation. Additionally, the devices differed in the suggested IOL, particularly when looking at calculations based on K-values taken from a corneal topographer, and when the study group was divided to low and moderate-high cylinder sub-groups. While these differences may not technically be considered clinically significant, they are worth consideration as the world progresses towards minimal residual post-operative refraction. Further investigation is required in order to assess the clinical impact on post-cataract surgery patients.

## Data Availability

The data set generated during the current study is available from the corresponding authors on reasonable request.
